# Diversity and Distribution of Mites (ACARI) Revealed by Contamination Survey in Public Genomic Databases

**DOI:** 10.3390/ani13203172

**Published:** 2023-10-11

**Authors:** Jiazheng Xie, Yi Zhang

**Affiliations:** Chongqing Key Laboratory of Big Data for Bio Intelligence, Chongqing University of Posts and Telecommunications, Chongqing 400065, China

**Keywords:** Acari, genomic contamination, diversity, distribution, evolution, DNA barcode

## Abstract

**Simple Summary:**

Mites are a group of minute animals ubiquitously distributed on the planet. They have close ecological ties with other species, such as plants, insects and vertebrates. With the development of sequencing technology, the genomic data have increased dramatically. Although the contaminations of microbial symbionts in public genomic databases have been explored to reveal the interactions between microbes and hosts, no similar study has been carried out to the microscopic mites. Here, we present a survey and analysis of the contamination of mites in Genbank genomic resources for the first time. The results showed that contamination of mites in public databases is not rare. Based on these contaminated contigs, the host associations and evolution of mites are discussed.

**Abstract:**

Acari (mites and ticks) are a biodiverse group of microarthropods within the Arachnida. Because of their diminutive size, mites are often overlooked. We hypothesized that mites, like other closely related microorganisms, could also contaminate public genomic database. Here, using a strategy based on DNA barcodes previously reported, we scanned contaminations related to mites (Acari, exclusive of Ixodida) in Genbank WGS/TSA database. In 22,114 assemblies (17,845 animal and 4269 plant projects), 1717 contigs in 681 assemblies (3.1%) were detected as mite contaminations. Additional taxonomic analysis showed the following: (1) most of the contaminants (1445/1717) were from the specimens of Magnoliopsida, Insecta and Pinopsida; (2) the contamination rates were higher in plant or TSA projects; (3) mite distribution among different classes of hosts varied considerably. Additional phylogenetic analysis of these contaminated contigs further revealed complicated mite-host associations. Overall, we conducted a first systemic survey and analysis of mite contaminations in public genomic database, and these DNA barcode related mite contigs will provide a valuable resource of information for understanding the diversity and phylogeny of mites.

## 1. Introduction

Acari (mites and ticks) are a highly speciose group of animals within the Arthropoda [1]. With nearly 55,000 described species and up to one million species awaiting discovery or description [2,3], mites can be found widely across various microhabitats around the world, from terrestrial to aquatic or oceanic environments, and even underground niches. Not surprisingly, their lifestyles are also highly diverse, from detritivorous, phytophagous, pollinivorous, fungivorous and predaceous in nonparasitic members to obligate ectoparasitism [1]. They have also multifaceted roles in ecosystems, such as pests of crops (e.g., spider mites and gall mites), parasites on birds and mammals (e.g., quill mites, scabies mites and follicle mites), vectors capable of transmitting notorious viruses and sources of allergens (e.g., house dust mites) [4]. Meanwhile, some of them can be beneficial to humans as biocontrol agents of pests and weeds. Although of great economic and ecological importance, our knowledge of mites is usually fragmentary which is focused on a particular mite taxon at a local scale [1,5], and many gaps still exist in our understanding of the distribution, diversification and evolution of mites.

The phylogenetic relationship among the main lineages of Acari was still a contentious issue [6,7,8]. In the current NCBI taxonomic system [9], Acari are comprised of two major lineages that have either monophyletic [10,11] or diphyletic origins [12]: the superorder Parasitiformes (Holothyrida + Ixodida (ticks) + Mesostigmata) and Acariformes (Trombidiformes + Sarcoptiformes). The Trombidiformes order contains a small suborder Sphaerolichida and a larger suborder Prostigmata which constists of three large clades (Eleutherengona, Anystina and Eupodina), and the Sarcoptiformes order includes three suborders (Endeostigmata, Oribatida and Astigmata).

Microbiologists have long been aware of contaminations in genomic databases caused by symbiotic bacteria, fungi or protists, and have utilized them as treasures to study the host-microbe interactions [13,14,15,16,17]. However, contaminations of the microscopic mites in genomic databases have not been studied. Our assumption is as follows: the ubiquitous mites, with very small size (mostly 0.4–0.8 mm) [2] and close associations to plants/animals, may go unnoticed in the field samples and have contaminated the public databases. Thus, we modified our previously published pipeline for protistan contaminations to survey mite contaminations in Genbank whole genome shotgun (WGS) genomes and transcriptome shotgun assemblies (TSA) based on DNA barcodes. DNA barcodes (e.g., the mitochondrial cytochrome c oxidase I, COI) are usually used in DNA barcoding experiments because such short sequences can produce accurate species identifications [18]. Our pipeline took advantage of this attribute, and was reliable to detect contaminations related to DNA barcodes in large genomic databases [13].

The aims of current study were as follows: (1) survey possible contaminations of mites in animal and plant genomic data; (2) compare the contamination rates between different sequencing methods (WGS against TSA), or among specimens of different host classes; (3) assess the various host associations of different mites, by calculating the distribution of mite contaminations among different host classes; (4) explore the phylogenetic origins of these contaminated contigs. Given the wide geographic scope and the breadth of organisms covered by Genbank WGS/TSA genomic database, we expect our findings will provide a broad illustration of the distribution and biodiversity of mites.

## 2. Materials and Methods

### 2.1. Database Retrieval

A total of 14,523 WGS and 7591 TSA assemblies within the taxonomic groups of “Metazoa (Animals) or Embryophyta (Land Plants), but not Acari” were downloaded from Genbank [19] (https://www.ncbi.nlm.nih.gov/Traces/wgs, accessed on 30 June 2023) (Information listed in Spreadsheet S1). Among them, there are 17,845 animal and 4269 plants assemblies, with 2.39 billion contigs (16 trillion bp).

The nonredundant BLAST nucleotide (or Genbank nt) database was downloaded from (https://ftp.ncbi.nlm.nih.gov/blast/db/, accessed on 27 December 2022).

The Barcode of Life Data System (BOLD) database [20], was downloaded from (http://www.boldsystems.org/index.php/datapackages, accessed on 7 July 2023). It includes 9,401,906 DNA barcodes from 9,090,674 specimens.

### 2.2. Pipeline of Mite Contamination Survey

**Figure 1:**
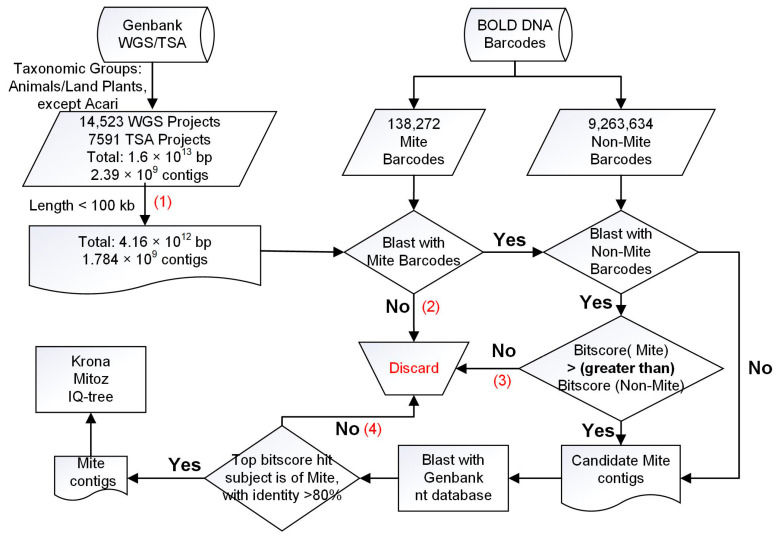
Pipeline to scan mite contamination. The four steps (1–4) to eliminate candidate sequences are marked in red font.

We modified our pipeline designed for scanning protistan contamination [13] by using mite barcodes as inclusion set and nonmite barcodes as exclusion set to scan mite contaminations (Figure 1). As Genbank WGS/TSA database is too large to be analyzed routinely, we sequentially eliminated candidate sequences by four steps that (1) were too long (>100,000 bp); (2) have no similarity to mite barcodes; (3) have more similarity to nonmite barcodes; (4) aligned with the best hit outside of Acari (exclusive of Ixodida) in the Genbank nt database, or with less than 80% identity.

Considering the huge size of Genbank WGS/TSA and the limitation of computational resources, we filtered contigs more than 100 kb based on the reason that all RefSeq mitochondrial genomes of the Acari are less than 25 kb (Figure S1a), and 98.5% of mite barcodes in BOLD library are COI related (Section 3.1 presents the detail); therefore, most of detected mite contaminations were mitochondrial-derived and shorter than 100 kb (Figure S1b).

### 2.3. Taxonomic Analysis of Mite Contaminated Contigs

To correctly assign the mite contaminated contigs to family, genus or even species level, the thresholds need be more restrictive. It has been reported that the DNA barcodes enable family taxonomic assignments in the Acari with strict similarity thresholds (Sarcoptiformes 89.9%, and Trombidiformes 91.4%) [21]. Thus, we further assigned the output contaminated contigs with mite origin to family level with a similarity threshold of 91.4%, according to the top best-score hit against nt database. The abundance of contaminated contigs was further plotted by Krona [22]. Additionally, the relative abundances were calculated as the percentages of contaminations with different mite family origins across different host classes, and plotted by means of the matplotlib library.

### 2.4. Phylogenetic Analysis of Contaminated Contigs

The phylogenetic markers COI were predicted with MitoZ [23]. The predicted COI with length more than 80 amino acids, plus reference sequences retrieved from GenBank (Table S1), were aligned with MAFFT v7.310 with the following option: mafft -maxiterate 10,000. The maximum likelihood (ML) tree was generated by IQ-tree V2.0.3 [24] with ultrafast bootstrap (UFBoot) [25] setting, and the following options: iqtree -m MFP -B 1000 -alrt 1000. The best-fit model according to Bayesian information criterion (BIC) score was mtInv+R7 [26]. The velvet spider *Stegodyphus mimosarum* [27] and Manchurian scorpion *Mesobuthus martensii* [28] were used as outgroups [11,29]. Phylogenetic tree was edited with FigTree V1.44 (https://github.com/rambaut/figtree/, accessed on 27 December 2022). All analyses were run on a high-performance computer server with dual Intel Xeon Platinum 8375C CPUs and 512 GB RAM.

## 3. Results

### 3.1. Mite DNA Barcodes in BOLD Database

Using a Python script with a regular expression (‘.*\|Animalia,Arthropoda,Arachnida,(Trombidiformes|Sarcoptiformes|Mesostigmata|Holothyrida)’) to match the sequence id, 138,272 DNA barcodes belonging to mites were extracted from the BOLD database to form the inclusion set, and the rest nonmite barcodes were used to build the exclusion set.

**Figure 2:**
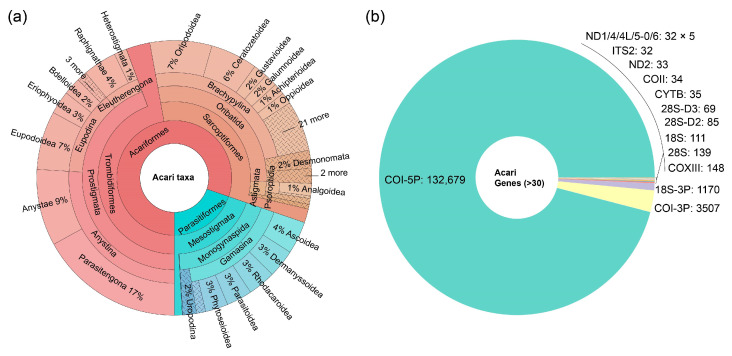
(**a**) Krona plot displaying the distribution of mite DNA barcodes at various Acari taxonomic levels in BOLD database. (**b**) Pie chart of mite DNA barcodes to different gene markers in BOLD database.

To get a better understanding of these mite barcodes, we plot the percentage of these barcodes by mite taxa (Figure 2a), and by genes (Figure 2b). The distribution of barcodes among mite taxa is as follows: Trombidiformes (65,183, 47%), Sarcoptiformes (45,819, 33%), Mesostigmata (27,268, 20%) and Holothyrida (2, 0%). The ratios are congruent with the numbers of described species in the taxa constituting the subclass Acari: i.e., Trombidiformes (25,797); Sarcoptiformes (16,299); Mesostigmata (11,424) and Holothyrida (27) [2].

As for the distribution among genes, COI-5P (132,679, 96%) plus COI-3P (3507, 2.5%) account for 98.5% of all the barcodes. The COI has long been used to discriminate the small mites, and to resolve the diversity of mite fauna in large-scale surveys [30,31]. It can overcome the shortage of external diagnostic characters of mites in traditional identification through morphology [32,33].

### 3.2. Mite Contaminations in Genbank nt Database

A substantial fraction of sequences in Genbank database appear to be contaminated [34]. Undetected mite contaminations in the Genbank nt database would lead to false negatives in the fourth step (Figure 1) of eliminating candidate sequences. However, our pipeline [13] could discriminate mite contaminations in the nt database, by checking those records that have 100% identity in the best match against misidentified sequences from the source species, but with the second-best match to mite sequences.

After running the pipeline, it output four misidentified sequences (mite contaminants) (Table 1) in the Genbank nt database. XM_022085578.1–XM_022085580.1 are annotated to be mitochondrial genes of *Zootermopsis nevadensis* (Dictyoptera, Termopsidae), but actually they are contaminations derived from the Acaroidea mite; and XR_002707260.1 is predicted to *Onthophagus taurus* small subunit rRNA, but the real source of this sequence is the Macrochelidae mite. Thus, we must be careful when using *COI*-like genes with the ‘-like’ suffix to identify species, because these genes are likely to be contaminants propagated from contaminations in Genbank WGS database.

### 3.3. Distribution of Mite Contaminations in Genbank WGS/TSA

In 22,114 assemblies (14,523 WGS and 7591 TSA projects), our modified pipeline resulted 1717 mite contaminated contigs (Figure S2, Fastafile S1) in 681 assemblies (220 WGS and 461 TSA projects). Thus, the contamination rate of TSA (6.1%) is higher than that of WGS (1.5%).

**Figure 3:**
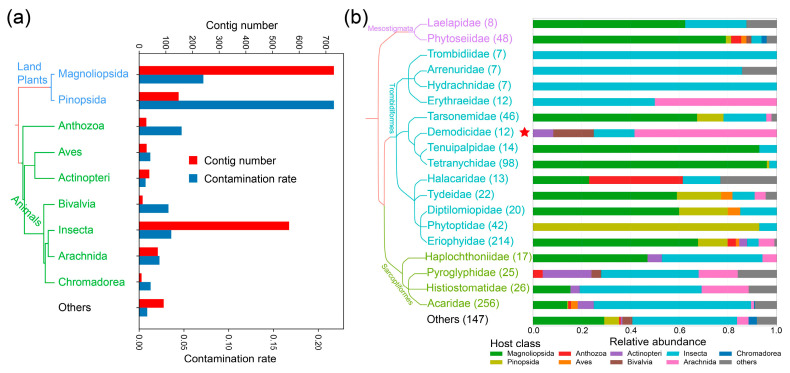
Distribution of mite contaminations among different host classes or mite families (**a**). Numbers of mite contigs or contamination rates among projects of different host classes. (**b**) Relative richness according to the percentages of contigs of different host classes to different mite families. The contig numbers are list in parentheses, and the host classes were indicated at the bottom of the plot. The host/mite cladogram trees were generated by taxtree (https://github.com/nongxinshengxin/taxtree, accessed on 6 August 2023) based on NCBI taxonomy. The artificial contamination with human *Demodex* (Demodicidae) is marked with a star symbol.

Next, we calculated the mite contig numbers, and contamination rates in specimens from different hosts (Figure 3a). The results showed that the richness of contaminations varied greatly among different host classes. The top three host classes with the largest number of contaminated contigs were as follows: Magnoliopsida (730 contigs), Insecta (562 contigs) and Pinopsida (148 contigs). Although the contamination rates of Pinopsida (30/138) and Magnoliopsida (290/4047) were higher than average (681/22,114), contamination rate of Insecta was not (223/6224).

To further reveal the distribution of mites, we assigned these contigs to mite families and plotted the relative abundance among different host classes (Figure 3b). Using a similarity threshold of 91.4%, 1041 contigs were successfully assigned to mite families. The distribution can be concluded as follows:

Contaminations in the order Mesostigmata are mostly from plant or insect specimens. For example, in the family Phytoseiidae which harbors most common plant inhabiting predatory mites [35], 38/48 of contaminated contigs are from projects of Magnoliopsida.

In the hyporder Parasitengona (Trombidiformes, Anystina), insect specimens are the predominant sources of contamination, although just a few contigs were detected in the following four families: Trombidiidae (7 contigs), Arrenuridae (7 contigs), Hydrachnidae (7 contigs) and Erythraeidae (12 contigs). Interestingly, in the family Erythraeidae, half of the contigs are from Arachnida assemblies. This is consistent with the reports that Parasitengona larvae can parasite on arthropods, such as larvae of Erythraeidae parasitic on spiders (Arachnida, Araneae) [36] and Harvestmen (Arachnida, Opiliones) [37].

For families in Eleutherengona, the detected contigs are modest: Tarsonemidae (46 contigs), Demodicidae (12 contigs), Tenuipalpidae (14 contigs) and Tetranychidae (98 contigs). Apart from Demodicidae, contaminations of these families are mostly associated with the class Magnoliopsida. Tetranychidae (spider mites) and Tenuipalpidae (false spider mites) are phytophagous and include major agricultural pests, thus are mainly found on plants. In the family Tarsonemidae (white mites), *Steneotarsonemus spinki* Smiley (rice mite) is a serious pest of rice crops, whereas some other genus/species are found associated with bark beetles [38,39]. We here found a modest percentage of contigs from Pinopsida and Insecta in Tarsonemidae. Demodicidae mites are ubiquitous skin parasites in mammals [40]. However, all 12 Demodicidae contigs here were related to nonmammal. After carefully checking these contigs, we found that all of them had high identities (96–100%) to the human mites (*Demodex folliculorum* or *Demodex brevis*) (Table S2); thus, we regard these Demodicidae contigs as fortuitous contaminations by human *Demodex* mites, and they should not be considered for further mite–host association analysis.

As for the supercohort Eupodina, (Diptilomiopidae (20 contigs) + Eriophyidae (214 contigs) + Phytoptidae (42 contigs) + Tydeidae (22 contigs) + Halacaridae (13 contigs)), most of them are phytophagous; thus, vagrant on host plants. Hence, most of the contaminants of Eupodina are found in assemblies of plants, except in the Halacaridae family. Notably, there were about 40% Halacaridae (marine mites) contigs from Anthozoa; and over 90% of Pinopsida in Phytoptidae.

Finally, in the order Sarcoptiformes, the most numerous of these contaminations were related to Insecta, followed by plants. Interestingly, of these, there are several contigs from the Actinopteri (bony fishes) assemblies (Table S3). This is consistent with the report that Histiostomatidae mites can attack fishes [41].

### 3.4. Phylogenetic Analysis of the Mite Contaminants

**Figure 4:**
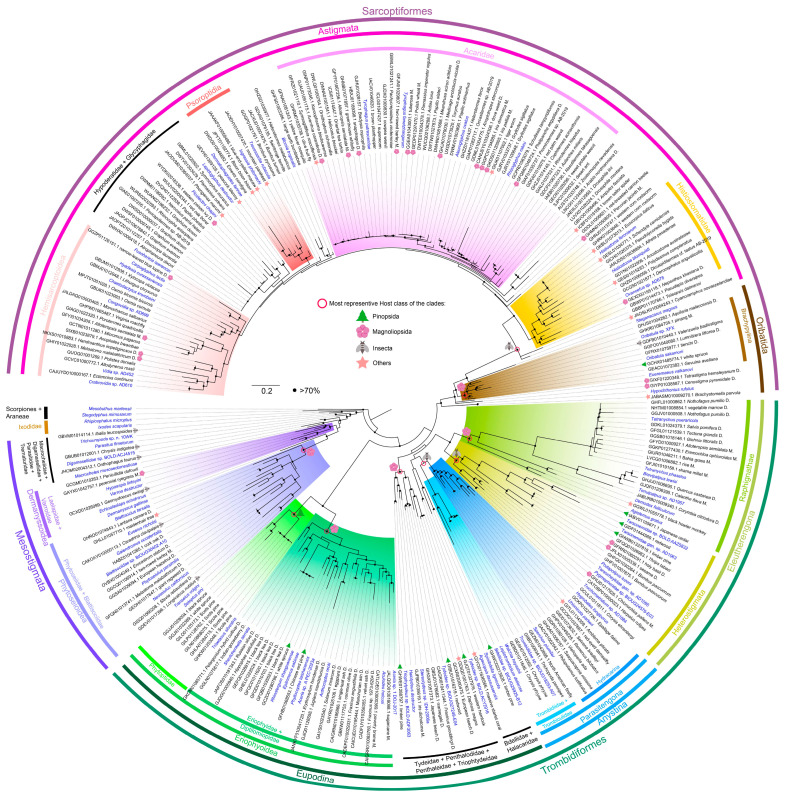
Phylogenetic tree of COI predicted from mite contaminated contigs. The species names of the mite references retrieved from Genbank were colored in blue font. The contaminated WGS/TSA contigs were named with accession numbers following the host names, with host classes represented by a symbol in the nodes (most representative class of that clade), or symbols after exceptional branches individually. The D. following names indicates the host taxon is dicots, and M. indicates monocots. Nodes with bootstrap values (BSP) ≥ 70% are marked with a black dot.

To further understand the phylogenetic origins of these contaminants, the contigs were annotated with MitoZ, and the predicted COI with a length more than 80 amino acids were used to infer a phylogenetic tree (Figure 4). The clades are colored according to the taxa of mite references retrieved from Genbank, and the host taxa of the contigs are derived from the project/assembly information (Spreadsheet S1) and indicated with symbols. As the preceding subsection revealed, similar host–mite associations can also be deduced from this smaller COI dataset.

According to the phylogenetic tree, conclusions can be drawn as follows: (1) the supercohort Anystina is monophyletic with low support, whereas the Eupodina is paraphyletic; (2) two superfamilies, Phytoseioidea (Blattisociidae + Phytoseiidae) and Eriophyoidea (Eriophyidae + Diptilomiopidae + Phytoptidae), were both recovered as monophyletic; (3) the monophylies of two clades, Parasitengona (Anystina) and Eleutherengona were also observed, but with low support in the clade of Parasitengona; (4) a monophyletic Hydracarina (Parasitengona) is strongly supported; (5) a close phylogenetic relationship of Parasitengona to a clade uniting Halacaridae and terrestrial predacious superfamily Bdelloidea was observed; (6) we also observed astigmatid mites nested in oribatid mites. These are consistent with the phylogenetic relationships of major mite groups reported before [8,12,42,43,44,45,46,47].

Next, we investigated the contamination by clades as follows:

Manure-inhabiting (Coprophilous) Mesostigmata mites are important biological control agents of pests that feed on the eggs or larvae of pests [48]. In Dung Beetles (*Onthophagus taurus*), a contig (JHOM02004312.1) was found related to the Macrochelidae (Mesostigmata) mite. And in this assembly, there was another contig related to rRNA (JHOM02004223.1) which was misidentified (XR_002707260.1) in the nt database (Table 1).

In Eriophyoidea clade [49], the hosts of contaminations can be divided into two groups: the dominant Magnoliopsida (angiosperms) (21/23) in the clade of (Eriophyidae + Diptilomiopidae), and Pinopsida (gymnosperms) (7/7) in Phytoptidae [49]. Interestingly, consistent with multiple host shift reported previously [8], in the clade of (Eriophyidae + Diptilomiopidae), there were two contigs from Pinopsida (gymnosperms) (GCZO01 and GFHB01) and a clade of monocots (JALQSO01 and CATLOE01), which are phylogenetically closest to mites that found in monocots before [50].

As for the aquatic mites, we found a contig (GIYO01) in massive starlet coral (Anthozoa) to Halacaridae clade. It has been reported that cold water coral reefs harbour a diverse Halacaridae fauna [51]. In the Hydracarina (Water mites) clade, there are four contigs from stoneflies (Plecoptera): *Setvena bradleyi* (GIEI01), *Remenus bilobatus* (GHPV01), *Viehoperla ada* (GIDP01) and *Sasquacapnia missiona* (GHQA01); two contigs from damselflies (Odonata, Zygoptera): *Epallage fatime* (GCKP01) and red-eyed damselfly (GCCK01); and one contig from caddisflies (Trichoptera): *Philopotamus ludificatus* (GACV01). The three orders Plecoptera, Odonata and Trichoptera are three major aquatic insect taxa [52]. This is consistent with the lifestyle of Hydracarina that harvest larvae and parasitize adults of aquatic insects [53]. Interestingly, one contig from *Amblema plicata* (Mollusca, GITL01) is closest to *Unionicola parkeri* mite (Hydracarina, Hygrobatoidea, Unionicolidae), which is a common symbiont of molluscs, by living on the gills or mantle and foot of their hosts [54,55].

In the Tetranychoidea (Eleutherengona, Raphignathae) clade, all the contaminated contigs are from Magnoliopsida; among them, the ratio of dicots to monocots is 8:6. There were two clades of Demodicidae (Raphignathae) and Stigmaeidae (Raphignathae) close to the Tetranychoidea. In the Demodicidae clade, the contig is from the black howler monkey (GGWL01), with 83.4% nucleotide identity to *Demodex folliculorum* (Table S2), a known mite parasite that inhabits the skin of humans [40]. In Stigmaeidae, it was a contig from Japanese cedar (Pinopsida; IABV01).

Oribatida are primarily soil dwelling, but also occur on trees [56]. For example, Eremaeidae *Eueremaeus trionus* was found on bases of branches of Siberian pine trees (*Pinus sibirica*) [57]. Thus, in the clade of Oribatida, we found most of the contigs are from Magnoliopsida (7/10). Interestingly, there was a contig from *Brachystomella parvula* (Collembola, JABASM01) which is closest to *Hypochthonius rufulus* (Oribatida, Hypochthoniidae). Springtails (Collembola) are also microarthropods that live below ground as Oribatida mites, and they are usually used together to reveal effects of the environmental change on soil microarthropod populations [58].

In Astigmata clade, the contigs are mostly sourced from Insecta or Magnoliopsida, except in Analgoidea (Psoroptidia). In Psoroptidia clade, there are two contigs from Ave: the mountain parakeet (JAOEHY01) and the blue-and-yellow macaw (JAAAKF01), and one contig from Mammalia: *Bison bison* (JPYT01). They are closest to the feather mite (Analgoidea) *Ingrassia philomachi* or *Dermatophagoides farinae* [59] in the phylogenetic tree.

Histiostomatoidea are typically associated with wet environments, and believed to be the earliest derivative Astigmata [45]. In Histiostomatoidea clade, the contigs are most from Insecta (8/12). The exceptions are as follows: contigs from *Euscorpius italicus* (Scorpiones; GKBL01), *Schendyla carniolensis* (Chilopoda; GESL01), *Polydesmus complanatus* (Diplopoda; GESI01) and *Nepenthes khasiana* (Magnoliopsida; GEXD02).

## 4. Discussion

Distribution and host associations of mites are complex because of their remarkable diversity of trophic preferences and habitats. Moreover, crossovers often occur (e.g., predators may feed on plants; free-living mites switch to parasitic or phoretic on other animals; and litter-inhabiting mites move onto plants) [1]. Thus, it is very challenging to summarise the distribution and host-interactions of mites.

Fundamental advances in sequencing technology and bioinformatics made en masse biodiversity assessments of microscopic organisms possible [60]. In this study, we applied a bioinformatics method to excavate mite contaminations in Genbank WGS/TSA database with acceptable computational costs and draw some conclusions that are in line with our expectations and mite–host associations concluded in traditional studies. However, we would like to emphasize some limitations of our study:

First, this study was not intended to survey all contaminated contigs related to all mite genes, but just those related to DNA barcodes. The reason was that the huge size and rapid growing of the Genbank database surpasses the limit of our computational resources, as we mentioned before [13].

Second, the mite contaminations detected by this study still have biases. The greatest number of mite species is found in soils [61]. However, we detected relatively few contigs of Oribatida and Endeostigmata (many of which live in deep soil). The reason is that Genbank WGS/TSA does not contain soil environmental data. Besides, the environmental specimens are not suitable for host association study because of the obscure host information.

Third, although BOLD barcode library is largely complete for vertebrate species, it remains poorly developed for invertebrates, especially mites [62]. Since our pipeline relied heavily on the BOLD and Genbank nt databases, we suppose there are still undetected mite contaminations related to unrecognized species. As the BOLD database is growing, it will provide sufficiently available barcodes to allow more precise resolution of the contaminated mites.

Lastly, as mites are so speciose, the contaminated contigs detected in this study still cannot cover all mite or host taxa. Hence, there are some mite families or host classes missed in our deduced distribution pattern. However, as the Genbank database growing, the mite contaminations will increase, and would provide more comprehensive information for mite distribution study.

## 5. Conclusions

In this study, we systematically studied the mite distribution based on contaminations in the Genbank WGS/TSA database, which covered a large cohort of species (animals: 10,240; plants: 1970; Spreadsheet S1). The results suggest that mite-derived contaminations are common in genomic databases, with three in a hundred of assemblies contaminated by mites. Thus, apart from commonly known microbial contaminations, we should also be aware of the contaminations derived from minuscule mites to avoid erroneous interpretation of the genomic data. Based on these valuable contaminated contigs, host associations of mites were concluded, such as Parasitengona mites on arthropods and Phytoseiidae, Tetranychidae, Tenuipalpidae and Eriophyoidea on plants. Further phylogenetic analysis of the predicted COI derived from these contigs corroborated the mite origination and heterogeneous distribution of the contaminated contigs. Overall, our study provides valuable insights into the global biodiversity and distribution of mites.

## Figures and Tables

**Table 1:** Misidentified sequences in Genbank nt database, which are actually sourced from mites.

Accession No ^1^ (WGS Prefix)	Matched Subject (Identity)	Len ^2^	Description of Subject Sequence ^3^
XM_022085578.1 (AUST01)	XM_022085578.1 (100)	1731	PREDICTED: *Zootermopsis nevadensis COX1*-like (LOC110840501), mRNA
	MN857505.1 (80.977)	1719	*Tyrophagus putrescentiae* voucher UMMZ BMOC 17-0108-002 mitochondrion, complete genome
XM_022085579.1 (AUST01)	XM_022085579.1 (100)	1321	PREDICTED: *Zootermopsis nevadensis COX3*-like (LOC110840502), mRNA
	MW784238.1 (77.51)	1245	*Lardoglyphus konoi* mitochondrion, complete genome
XM_022085580.1 (AUST01)	XM_022085580.1 (100)	760	PREDICTED: *Zootermopsis nevadensis COX2*-like (LOC110840503), mRNA
	NC_038058.1 (81.659)	687	*Rhizoglyphus robini* mitochondrion, complete genome
XR_002707260.1 (JHOM02)	XR_002707260.1 (100)	1790	PREDICTED: *Onthophagus taurus* Eukaryotic small subunit rRNA (LOC111421936)
	AY620939.1 (97.452)	1766	*Macrocheles* sp. AL5995 18S rRNA gene, partial sequence

^1^ The misidentified sequences in the Genbank nt database were blasted against nt database; the top two best matches were listed, with the first record to itself and the second to mite sequence. ^2^ Alignment length. ^3^ Abbreviation: ‘cytochrome c oxidase subunit’, *COX*; ‘ribosomal RNA’, rRNA.

## Data Availability

The Genbank WGS/TSA datasets for this study can be downloaded in [GenBank] (https://www.ncbi.nlm.nih.gov/GenBank, accessed on 30 June 2023). The Barcode of Life Data System library can be found in [Bold] (http://www.boldsystems.org/index.php/datapackages, accessed on 7 July 2023). The bioinformatic code is available at (https://github.com/xiebio/DBCscan, accessed on 13 August 2023).

## Supplementary Materials

The following supporting information can be downloaded at: https://www.mdpi.com/article/10.3390/ani13203172/s1, Figure S1: Length distribution of (a) 59 mitochondrion genomes of mites in Refseq database, and (b) 1717 mite contaminated contigs in Genbank WGS/TSA database detected in this study; Figure S2: Relative abundance of mite contaminated contigs at various Acari taxonomic levels detected in Genbank WGS/TSA database; Table S1: List of protein sequences retrieved from Genbank for the phylogenetic analysis; Table S2: Contaminations of family Demodicidae; Table S3: Contaminations related to the order Sarcoptiformes in fish (Actinopteri) assemblies; Spreadsheet S1: WGS & TSA assembly info.xls; Fastafile S1: Mite contaminated contigs.fasta.

## References

[1] Krantz G.W., Walter D.E. (2009). A Manual of Acarology.

[2] Stork N.E. (2018). How Many Species of Insects and Other Terrestrial Arthropods Are There on Earth?. Annu. Rev. Entomol..

[3] Zhang Z.-Q. (2011). Animal Biodiversity: An Outline of Higher-Level Classification and Survey of Taxonomic Richness.

[4] Hammad H., Chieppa M., Perros F., Willart M.A., Germain R.N., Lambrecht B.N. (2009). House dust mite allergen induces asthma via Toll-like receptor 4 triggering of airway structural cells. Nat. Med..

[5] Gan H., Zak D.R., Hunter M.D. (2019). Scale dependency of dispersal limitation, environmental filtering and biotic interactions determine the diversity and composition of oribatid mite communities. Pedobiologia.

[6] Xue X.-F., Dong Y., Deng W., Hong X.-Y., Shao R. (2017). The phylogenetic position of eriophyoid mites (superfamily Eriophyoidea) in Acariformes inferred from the sequences of mitochondrial genomes and nuclear small subunit (18S) rRNA gene. Mol. Phylogenetics Evol..

[7] Klimov P.B., Oconnor B.M., Chetverikov P.E., Bolton S.J., Pepato A.R., Mortazavi A.L., Tolstikov A.V., Bauchan G.R., Ochoa R. (2018). Comprehensive phylogeny of acariform mites (Acariformes) provides insights on the origin of the four-legged mites (Eriophyoidea), a long branch. Mol. Phylogenetics Evol..

[8] Xue X.F., Yao L.F., Yin Y., Liu Q., Li N., Hoffmann A.A., Sun J.T., Hong X.Y. (2023). Macroevolutionary analyses point to a key role of hosts in diversification of the highly speciose eriophyoid mite superfamily. Mol. Phylogenetics Evol..

[9] Federhen S. (2012). The NCBI Taxonomy database. Nucleic Acids Res..

[10] Lozano-Fernandez J., Tanner A.R., Giacomelli M., Carton R., Vinther J., Edgecombe G.D., Pisani D. (2019). Increasing species sampling in chelicerate genomic-scale datasets provides support for monophyly of Acari and Arachnida. Nat. Commun..

[11] Zhang Y.-X., Chen X., Wang J.-P., Zhang Z.-Q., Wei H., Yu H.-Y., Zheng H.-K., Chen Y., Zhang L.-S., Lin J.-Z. (2019). Genomic insights into mite phylogeny, fitness, development, and reproduction. BMC Genom..

[12] Pepato A.R., Costa S.G.d.S., Harvey M.S., Klimov P.B. (2022). One-way ticket to the blue: A large-scale, dated phylogeny revealed asymmetric land-to-water transitions in acariform mites (Acari: Acariformes). Mol. Phylogenetics Evol..

[13] Xie J., Tan B., Zhang Y. (2023). A Large-Scale Study into Protist-Animal Interactions Based on Public Genomic Data Using DNA Barcodes. Animals.

[14] Orosz F. (2023). Presence of p25alpha-Domain in Seed Plants (Spermatophyta): Microbial/Animal Contaminations and/or Orthologs. Life.

[15] Twort V.G., Blande D., Duplouy A. (2022). One’s trash is someone else’s treasure: Sequence read archives from Lepidoptera genomes provide material for genome reconstruction of their endosymbionts. BMC Microbiol..

[16] Borner J., Burmester T. (2017). Parasite infection of public databases: A data mining approach to identify apicomplexan contaminations in animal genome and transcriptome assemblies. BMC Genom..

[17] Lopes R.J., Merida A.M., Carneiro M. (2017). Unleashing the Potential of Public Genomic Resources to Find Parasite Genetic Data. Trends Parasitol..

[18] Min X.J., Hickey D.A. (2007). DNA Barcodes Provide a Quick Preview of Mitochondrial Genome Composition. PLoS ONE.

[19] Benson D.A., Karsch-Mizrachi I., Lipman D.J., Ostell J., Sayers E.W. (2009). GenBank. Nucleic Acids Res..

[20] Ratnasingham S., Hebert P.D.N. (2007). BOLD: The Barcode of Life Data System (http://www.barcodinglife.org). Mol. Ecol. Notes.

[21] Young M.R., deWaard J.R., Hebert P.D.N. (2021). DNA barcodes enable higher taxonomic assignments in the Acari. Sci. Rep..

[22] Ondov B.D., Bergman N.H., Phillippy A.M. (2011). Interactive metagenomic visualization in a Web browser. BMC Bioinform..

[23] Meng G., Li Y., Yang C., Liu S. (2019). MitoZ: A toolkit for animal mitochondrial genome assembly, annotation and visualization. Nucleic Acids Res..

[24] Minh B.Q., Schmidt H.A., Chernomor O., Schrempf D., Woodhams M.D., von Haeseler A., Lanfear R. (2020). IQ-TREE 2: New Models and Efficient Methods for Phylogenetic Inference in the Genomic Era. Mol. Biol. Evol..

[25] Hoang D.T., Chernomor O., von Haeseler A., Minh B.Q., Vinh L.S. (2018). UFBoot2: Improving the Ultrafast Bootstrap Approximation. Mol. Biol. Evol..

[26] Kalyaanamoorthy S., Minh B.Q., Wong T.K.F., von Haeseler A., Jermiin L.S. (2017). ModelFinder: Fast model selection for accurate phylogenetic estimates. Nat. Methods.

[27] Johannesen J., Lubin Y., Smith D.R., Bilde T., Schneider J.M. (2007). The age and evolution of sociality in Stegodyphus spiders: A molecular phylogenetic perspective. Proc. R. Soc. B Biol. Sci..

[28] Choi E.H., Park S.J., Jang K.H., Hwang W. (2007). Complete mitochondrial genome of a chinese scorpion *Mesobuthus martensii* (Chelicerata, scorpiones, buthidae). DNA Seq..

[29] Liu Q., Deng Y., Song A., Xiang Y., Chen D., Wei L. (2021). Comparative analysis of mite genomes reveals positive selection for diet adaptation. Commun. Biol..

[30] Young M.R., Proctor H.C., deWaard J.R., Hebert P.D.N. (2019). DNA barcodes expose unexpected diversity in Canadian mites. Mol. Ecol..

[31] deWaard J.R., Ratnasingham S., Zakharov E.V., Borisenko A.V., Steinke D., Telfer A.C., Perez K.H.J., Sones J.E., Young M.R., Levesque-Beaudin V. (2019). A reference library for Canadian invertebrates with 1.5 million barcodes, voucher specimens, and DNA samples. Sci. Data.

[32] Yin Y., Yao L.-F., Hu Y., Shao Z.-K., Hong X.-Y., Hebert P.D.N., Xue X.-F. (2022). DNA barcoding uncovers cryptic diversity in minute herbivorous mites (Acari, Eriophyoidea). Mol. Ecol. Resour..

[33] Pérez-Sayas C., Pina T., Sabater-Muñoz B., Gómez-Martínez M.A., Jaques J.A., Hurtado-Ruiz M.A. (2022). DNA Barcoding and Phylogeny of Acari Species Based on ITS and COI Markers. J. Zool. Syst. Evol. Res..

[34] Steinegger M., Salzberg S.L. (2020). Terminating contamination: Large-scale search identifies more than 2,000,000 contaminated entries in GenBank. Genome Biol..

[35] Demite P.R., McMurtry J.A., De Moraes G.J. (2014). Phytoseiidae Database: A website for taxonomic and distributional information on phytoseiid mites (Acari). Zootaxa.

[36] Makol J., Felska M. (2011). New records of spiders (Araneae) as hosts of terrestrial Parasitengona mites (Acari: Actinotrichida: Prostigmata). J. Arachnol..

[37] Gabrys G., Felska M., Klosinska A., Starega W., Makol J. (2011). Harvestmen (Opiliones) as hosts of Parasitengona (Acari: Actinotrichida, Prostigmata) larvae. J. Arachnol..

[38] Karmakar K. (2008). Steneotarsonemus spinki Smiley (Acari: Tarsonemidae)—A yield reducing mite of rice crops in West Bengal, India. Int. J. Acarol..

[39] Khaustov A.A., Petrov A.V., Kolesnikov V.B. (2021). A new genus and two new species of Tarsonemidae (Acari: Heterostigmata) associated with bark beetles (Coleoptera: Curculionidae: Scolytinae) from Peru. Zootaxa.

[40] Palopoli M.F., Minot S., Pei D., Satterly A., Endrizzi J. (2014). Complete mitochondrial genomes of the human follicle mites Demodex brevis and D. folliculorum: Novel gene arrangement, truncated tRNA genes, and ancient divergence between species. BMC Genom..

[41] Halliday R.B., Collins R.O. (2002). *Histiostoma papillata* sp. n. (Acari: Histiostomatidae), a mite attacking fish in Australia. Aust. J. Entomol..

[42] Dabert M., Proctor H., Dabert J. (2016). Higher-level molecular phylogeny of the water mites (Acariformes: Prostigmata: Parasitengonina: Hydrachnidiae). Mol. Phylogenetics Evol..

[43] Pepato A.R., Klimov P.B. (2015). Origin and higher-level diversification of acariform mites–evidence from nuclear ribosomal genes, extensive taxon sampling, and secondary structure alignment. BMC Evol. Biol..

[44] Li W.-N., Shao R., Zhang Q., Deng W., Xue X.-F. (2019). Mitochondrial genome reorganization characterizes various lineages of mesostigmatid mites (Acari: Parasitiformes). Zool. Scr..

[45] Norton R.A. (1998). Morphological evidence for the evolutionary origin of Astigmata (Acari: Acariformes). Exp. Appl. Acarol..

[46] Li W.-N., Xue X.-F. (2019). Mitochondrial genome reorganization provides insights into the relationship between oribatid mites and astigmatid mites (Acari: Sarcoptiformes: Oribatida). Zool. J. Linn. Soc..

[47] Dabert M., Witalinski W., Kazmierski A., Olszanowski Z., Dabert J. (2010). Molecular phylogeny of acariform mites (Acari, Arachnida): Strong conflict between phylogenetic signal and long-branch attraction artifacts. Mol. Phylogenetics Evol..

[48] Farahi S., Shishehbor P., Nemati A., Perotti M.A. (2022). Mesostigmata diversity by manure type: A reference study and new datasets from southwestern Iran. Exp. Appl. Acarol..

[49] Li H.-S., Hoffmann A.A., Guo J.-F., Zuo Y., Xue X.-F., Pang H., Hong X.-Y. (2016). Identification of two lineages of host-associated eriophyoid mites predisposed to different levels of host diversification. Mol. Phylogenetics Evol..

[50] Chetverikov P.E., Fedorov D.S., Letukhova V.Y., Romanovich A.E. (2021). Description of Cecidophyes fibigiae n. sp., new combinations, records, and DNA barcodes of eriophyid mites (Eriophyoidea, Eriophyidae) from Karadag Nature Reserve (Crimea). Syst. Appl. Acarol..

[51] Bartsch I. (2020). Lohmannella (Acari, Halacaridae) from a cold-water coral reef off Norway, description of two new and a list of North Atlantic species. Zootaxa.

[52] Sanchez-Bayo F., Wyckhuys K.A.G. (2019). Worldwide decline of the entomofauna: A review of its drivers. Biol. Conserv..

[53] Vasquez A.A., Kabalan B.A., Ram J.L., Miller C.J. (2020). The Biodiversity of Water Mites That Prey on and Parasitize Mosquitoes. Diversity.

[54] Edwards D.D., Vidrine M.F., Ernsting B.R. (2010). Phylogenetic relationships among Unionicola (Acari: Unionicolidae) mussel-mites of North America based on mitochondrial cytochrome oxidase I sequences. Zootaxa.

[55] Edwards D.D., Jackson L.E., Johnson A.J., Ernsting B.R. (2011). Mitochondrial genome sequence of Unionicola parkeri (Acari: Trombidiformes: Unionicolidae): Molecular synapomorphies between closely-related Unionicola gill mites. Exp. Appl. Acarol..

[56] Schaffer S., Koblmuller S., Krisper G. (2020). Revisiting the Evolution of Arboreal Life in Oribatid Mites. Diversity.

[57] Salavatulin V. (2019). Microhabitat distribution of arboreal oribatid mites (Oribatida), associated with the Siberian pine (*Pinus sibirica*) of Western Siberia. Exp. Appl. Acarol..

[58] Zhu D., Bi Q.-F., Xiang Q., Chen Q.-L., Christie P., Ke X., Wu L.-H., Zhu Y.-G. (2018). Trophic predator-prey relationships promote transport of microplastics compared with the single *Hypoaspis aculeifer* and *Folsomia candida*. Environ. Pollut..

[59] Klimov P.B., Oconnor B.M. (2009). Improved tRNA prediction in the American house dust mite reveals widespread occurrence of extremely short minimal tRNAs in acariform mites. BMC Genom..

[60] Bik H.M., Porazinska D.L., Creer S., Caporaso J.G., Knight R., Thomas W.K. (2012). Sequencing our way towards understanding global eukaryotic biodiversity. Trends Ecol. Evol..

[61] Arribas P., Andujar C., Moraza M.L., Linard B., Emerson B.C., Vogler A.P. (2019). Mitochondrial metagenomics reveals the ancient origin and phylodiversity of soil mites and provides a phylogeny of the Acari. Mol. Biol. Evol..

[62] Trebitz A.S., Hoffman J.C., Grant G.W., Billehus T.M., Pilgrim E.M. (2015). Potential for DNA-based identification of Great Lakes fauna: Match and mismatch between taxa inventories and DNA barcode libraries. Sci. Rep..

