# Supplementary material for: Diversity and Distribution of Mites (ACARI) Revealed by Contamination Survey in Public Genomic Databases
Source: Animals (Basel). 2023 Oct 11;13(20):3172. doi: 10.3390/ani13203172 (PMC10603697; doi:10.3390/ani13203172)
Supplement: Supplementary file 1 [file animals-13-03172-s001.zip › Supplementary Materials/Supplementary figure and tables.pdf]

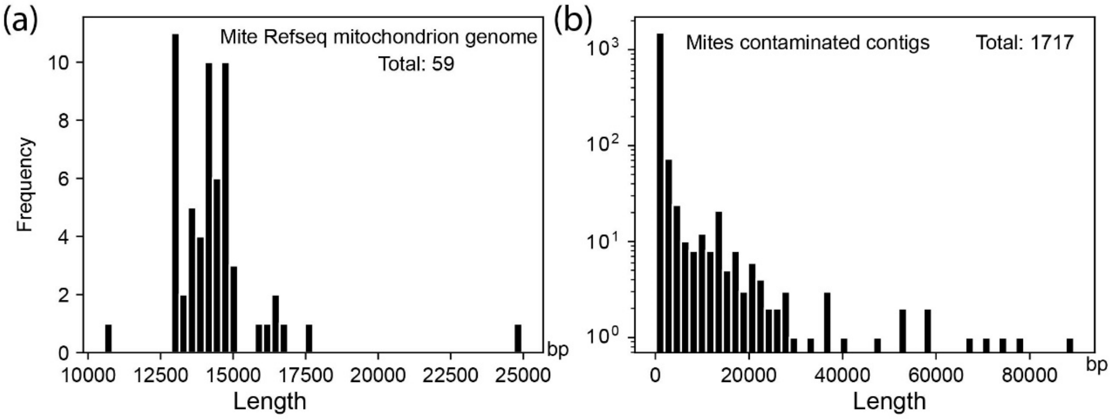

Figure S1. Length distribution of (a) 59 mitochondrion genomes of mites in Refseq database, and (b) 1717 mite contaminated contigs in Genbank WGS/TSA database detected in this study.

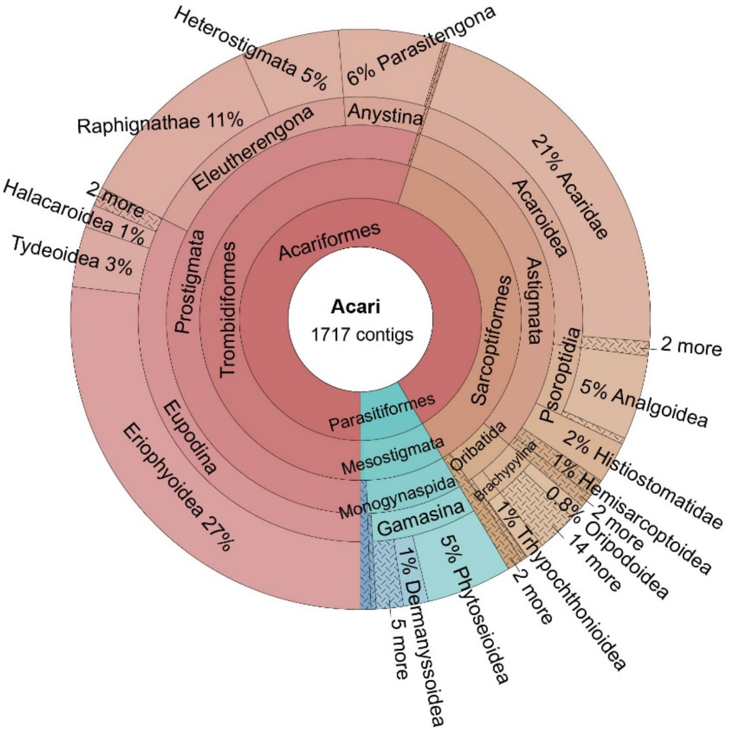

Figure S2. Relative abundance of mite contaminated contigs at various Acari taxonomic levels detected in Genbank WGS/TSA database.

Table S1. List of protein sequences retrieved from Genbank for the phylogenetic analysis.

Species	Hyporder/ Suborder/ Infraorder	Superfamily	Family	Accession
Hygrobates longiporus	Anystina	Hygrobatoidea	Hygrobatidae	YP_009866786.1
Sperchon plumifer	Anystina	Sperchontoidea	Sperchontidae	YP_009535749.1
Unionicola parkeri	Anystina	Hygrobatoidea	Unionicolidae	YP_004021457.1
Walchia hayashii	Anystina	Trombiculoidea	Trombiculidae	YP_001837111.1
Trombidiidae sp. BIOUG18253-A07	Anystina	Trombidioidea	Trombidiidae	AWS19674.1
Stegodyphus mimosarum	Araneomorphae	Eresoidea	Eresidae	ABJ96298.1
Lepidoglyphus destructor	Astigmata	Glycyphagoidea	Glycyphagidae	YP_010354348.1
Blomia tropicalis	Astigmata	Glycyphagoidea	Echimyopodidae	YP_010354335.1
Carpoglyphus lactis	Astigmata	Hemisarcoptoidea	Carpoglyphidae	YP_009862148.1
Trouessartia rubecula	Astigmata	Analgoidea	Trouessartiidae	YP_009560912.1
Histiostoma feroniarum	Astigmata	Histiostomatoidea	Histiostomatidae	YP_009504242.1
Rhizoglyphus robini	Astigmata	Acaroidea	Acaridae	YP_009498332.1
Histiostoma blomquisti	Astigmata	Histiostomatoidea	Histiostomatidae	YP_009306839.1
Tyrophagus putrescentiae	Astigmata	Acaroidea	Acaridae	YP_009114537.1
Aleuroglyphus ovatus	Astigmata	Acaroidea	Acaridae	YP_009019414.1
Dermatophagoides farinae	Astigmata	Analgoidea	Pyroglyphidae	YP_003162772.1
Ovanoetus sp. AD678	Astigmata	Histiostomatoidea	Histiostomatidae	ATY50495.1
Chaetodactylus krombeini	Astigmata	Hemisarcoptoidea	Chaetodactylidae	ATY50504.1
Crabrovidia sp. AD610	Astigmata	Hemisarcoptoidea	Winterschmidtiidae	ATY50500.1
Ingrassia philomachi	Astigmata	Analgoidea	Xolalgidae	AOO33910.1
Hyadesia curassaviensis	Astigmata	Hemisarcoptoidea	Hyadesiidae	ATY50501.1
Vidia sp. AD452	Astigmata	Hemisarcoptoidea	Winterschmidtiidae	ATY50499.1
Congovidia sp. AD699	Astigmata	Hemisarcoptoidea	Hemisarcoptidae	ATY50496.1
Fusohericia lawrencei	Astigmata	Hemisarcoptoidea	Algophagidae	ATY50503.1
Tyrophagus fanetzhangorum	Astigmata	Acaroidea	Acaridae	AVX48499.1
Picidectes chapini	Astigmata	Hypoderatoidea	Hypoderatidae	UJR87502.1
Oribatula sakamorii	Brachypylina	Oripodoidea	Oribatulidae	QYF07803.1
Oribatula sp. XFX	Brachypylina	Oripodoidea	Oribatulidae	QFG71641.1
Eueremaeus valkanovi	Brachypylina	Eremaeoidea	Eremaeidae	WFK33970.1
Demodex folliculorum	Eleutherengona	Cheyletoidea	Demodicidae	YP_009114835.1
Tetranychus pueraricola	Eleutherengona	Tetranychoidea	Tetranychidae	YP_009051530.1
Tetranychus phaselus	Eleutherengona	Tetranychoidea	Tetranychidae	YP_009051517.1
Tarsonemidae gen. sp. AD1063	Eleutherengona	Tarsonemoidea	Tarsonemidae	ATY50430.1
Trochometridium sp. AD1884	Eleutherengona	Pyemotoidea	Trochometridiidae	ATY50433.1
Agistemus gratus	Eleutherengona	Raphignathoidea	Stigmaeidae	WAK43286.1
Tarsocheylidae gen. sp. AD1095	Eleutherengona	Tarsocheyloidea	Tarsocheylidae	ATY50428.1
Podapolipus luzoni	Eleutherengona	Tarsonemoidea	Podapolipidae	ARI47195.1
Brevipalpus lewisi	Eleutherengona	Tetranychoidea	Tenuipalpidae	QAU55254.1
Pygmephoridae sp. BIOUG24576-E03	Eleutherengona	Pygmephoroidea	Pygmephoridae	AYN51884.1
Tarsonemidae sp. BOLD: AAZ5832	Eleutherengona	Tarsonemoidea	Tarsonemidae	AFW14558.1

Tenuipalpus sp. AD1957	Eleutherengona	Tetranychoidea	Tenuipalpidae	ATY50462.1
Hypochthonius rufulus	Enarthronota	Hypochthonoidea	Hypochthoniidae	UYZ39297.1
Halotydeus destructor	Eupodina	Eupodoidea	Penthaleidae	YP_010385508.1
Spinibdella lignicola	Eupodina	Bdelloidea	Bdellidae	YP_010516613.1
Rhinotergum shaoguanense	Eupodina	Eriophyoidea	Diptilomiopidae	YP_009351807.1
Phyllocoptes taishanensis	Eupodina	Eriophyoidea	Eriophyidae	YP_009228533.1
Epitrimerus sabinae	Eupodina	Eriophyoidea	Eriophyidae	YP_009228520.1
Agauopsis itassussensis	Eupodina	Halacaroidea	Halacaridae	UJR87586.1
Triophytydeus sp. BOLD:ADF3063	Eupodina	Tydeoidea	Triophtydeidae	QGW41996.1
Aculodes holcusi	Eupodina	Eriophyoidea	Eriophyidae	QQK89385.1
Setoptus pini	Eupodina	Eriophyoidea	Phytoptidae	QHB50645.1
Anthocoptes recki	Eupodina	Eriophyoidea	Eriophyidae	QQK89387.1
Trisetacus silvestris	Eupodina	Eriophyoidea	Phytoptidae	QHB50694.1
Bdellidae sp. BIOUG25925-B12	Eupodina	Bdelloidea	Bdellidae	AYN51502.1
Stereotydeus sp. IDH-2006a	Eupodina	Eupodoidea	Penthalodidae	ABD17886.1
Aceria sp. A PEC-2023a	Eupodina	Eriophyoidea	Eriophyidae	WIW35252.1
Tydeidae sp. BOLD:ACY2916	Eupodina	Tydeoidea	Tydeidae	QGQ51928.1
Tydeidae sp. BIOUG27249-E04	Eupodina	Tydeoidea	Tydeidae	AWK31052.1
Aceria sp. 1 DOJ-2017	Eupodina	Eriophyoidea	Eriophyidae	ASO96446.1
Shevtchenkella serrata	Eupodina	Eriophyoidea	Eriophyidae	UCS82606.1
Parasitus fimetorum	Gamasina	Parasitoidea	Parasitidae	YP_010321832.1
Neoseiulus californicus	Gamasina	Phytoseioidea	Phytoseiidae	YP_010584664.1
Dermanyssus gallinae	Gamasina	Dermanyssoidea	Dermanyssidae	YP_010045993.1
Hypoaspis linteyini	Gamasina	Dermanyssoidea	Laelapidae	YP_009731531.1
Blattisocius tarsalis	Gamasina	Phytoseioidea	Blattisociidae	YP_009731518.1
Macrocheles muscaedomesticae	Gamasina	Eviphidoidea	Macrochelidae	YP_009731505.1
Varroa destructor	Gamasina	Dermanyssoidea	Varroidae	NP_758874.1
Galendromus occidentalis	Gamasina	Phytoseioidea	Phytoseiidae	YP_001096000.1
Digamasellidae sp. BOLD:ACJ4515	Gamasina	Rhodacaroidea	Digamasellidae	ALS53963.1
Echinolaelaps echidninus	Gamasina	Dermanyssoidea	Laelapidae	WHL46305.1
Transeius volgini	Gamasina	Phytoseioidea	Phytoseiidae	QPZ18645.1
Phytoseiulus persimilis	Gamasina	Phytoseioidea	Phytoseiidae	ACS15261.1
Euseius nicholsi	Gamasina	Phytoseioidea	Phytoseiidae	AGX31987.1
Blattisociidae sp. BIOUG30402-A10	Gamasina	Phytoseioidea	Blattisociidae	AYK98228.1
Steganacarus magnus	Mixonomata	Phthiracaroidea	Steganacaridae	YP_002317272.1
Trichouropoda sp. n. 10WK	Uropodina		Trematuridae	AEW49442.1
Ixodes scapularis		Ixodoidea	Ixodidae	YP_010535455.1
Rhipicephalus microplus		Ixodoidea	Ixodidae	YP_008999527.1
Mesobuthus martensii		Buthoidea	Buthidae	YP_001427343.1

Table S2. Contaminations of family Demodicidae. 12 Contigs in red fonts with identities more than 96% are supposed to be artificial contaminations of human *Demodex* mites. The other 2 contigs (GGWL01055778.1 and GGWL01055779.1) from black howler monkey assembly (GGWL01) with identities less than 91.4% were not assigned to family level.

CONTIG ACCESSION	CONTIG LENGTH	ASSEMBLY HOST	BEST BLAST SUBJECT	IDENTITY	BLAST LENGTH	SUBJECT DESCRIPTION
GGWL01055778.1	1519	Mammalia	KM114226.1	83.433	1509	Demodex folliculorum mitochondrion, complete genome
GGWL01055779.1	302	Mammalia	OP900959.1	88.889	297	Demodex melesinus isolate SB11 cytochrome c oxidase subunit I (COX1) gene, partial cds; mitochondrial
JAODFX010125061.1	248	Insecta	KM114225.1	100	248	Demodex brevis mitochondrion, complete genome
HBDQ01152762.1	1190	Bivalvia	KM114226.1	96.057	989	Demodex folliculorum mitochondrion, complete genome
GHHL01022903.1	325	Arachnida		100	325	
GHHL01022904.1	360	Arachnida		96	350	
	360	Arachnida		100	58	Demodex folliculorum voucher UMMZ BMOC 07-0815-005 AD1011 18S ribosomal RNA gene, partial sequence
GHHL01022905.1	325	Arachnida		100	246	
	325	Arachnida	KY922187.1	100	86	
GHHL01034003.1	226	Arachnida		100	148	
	226	Arachnida		100	82	
GHHL01034004.1	208	Arachnida		100	167	
	208	Arachnida		98	50	
GHHL01075817.1	231	Arachnida		98.701	231	Demodex folliculorum voucher UMMZ BMOC 07-0815-005 AD1011 28S ribosomal RNA gene, partial sequence
GHHL01075818.1	218	Arachnida		98.165	218	
GKBK01103287.1	296	Insecta	KY922058.1	98.986	296	
HBDQ01066108.1	346	Bivalvia		98.83	342	
GKEF01806478.1	228	Actinopteri	MH540325.1	100	200	Demodex brevis isolate D3-1 28S ribosomal RNA gene, partial sequence

Table S3. Contaminations related to the order Sarcoptiformes in fish (Actinopteri) assemblies. Contaminated contigs were blasted against Genbank nt database, the best score hits were listed in the table.

CONTAMINATED CONTIG ACCESSION	ASSEMBLY HOST	BEST BLAST SUBJECT	IDENTITY	BLAST LENGTH	SUBJECT Family
JAODHT010034050.1	*Nemapteryx augusta*	KY922458.1	99.649	285	Haplochthoniidae
GDHT01210928.1	*Austrofundulus limnaeus*	JQ000249.1	100	224	
HALZ01069874.1	*Sardina pilchardus*	XR_006959345.1	95.11	409	
HAMA01003558.1	*Sardina pilchardus*	XR_003474832.1	99.153	236	Pyroglyphidae
HAMB01025712.1	*Sardina pilchardus*	XR_006959345.1	97.835	231	
HBYV01088761.1	*Platichthys flesus*	XR_006959345.1	100	205	
GIAU01075596.1	*Lucifuga dentata*	JQ000367.1	96.226	318	Histiostomatidae
GFMN01017841.1	*Chionodraco hamatus*	MN857506.1	99.62	526	
GIAU01155259.1	*Lucifuga dentata*	MN857505.1	100	246	
GIAU01165557.1	*Lucifuga dentata*	MN857506.1	98.529	408	
GIAU01192799.1	*Lucifuga dentata*	KM277806.1	98.026	304	
GIAU01192800.1	*Lucifuga dentata*	KM277806.1	96.711	304	
GIAU01202434.1	*Lucifuga dentata*	MN857506.1	100	254	
GIAU01367655.1	*Lucifuga dentata*	KM277818.1	99.694	327	
GIAU01367663.1	*Lucifuga dentata*	KM277818.1	96.697	333	
GIAU01399740.1	*Lucifuga dentata*	MK393792.1	100	206	Acaridae
GIAU01472509.1	*Lucifuga dentata*	MN857505.1	99.526	211	
GJOL01045947.1	*Cotylopus acutipinnis*	KM277806.1	99.825	570	
GKAU01101372.1	*Tenualosa ilisha*	MN857505.1	99.231	260	
HANK010104528.1	*Pleuronectes platessa*	KM277817.1	100	251	
HANK010201860.1	*Pleuronectes platessa*	KM277818.1	100	342	
HANK010573285.1	*Pleuronectes platessa*	KM277818.1	92.857	266	
HANK010592382.1	*Pleuronectes platessa*	MN857506.1	99.82	1114	
HANK010592383.1	*Pleuronectes platessa*	MN857506.1	100	548	
